# Dysphagia as Isolated Manifestation of Jo-1 Associated Myositis?

**DOI:** 10.3389/fneur.2019.00739

**Published:** 2019-07-09

**Authors:** Bendix Labeit, Paul Muhle, Sonja Suntrup-Krueger, Sigrid Ahring, Tobias Ruck, Rainer Dziewas, Tobias Warnecke

**Affiliations:** Department of Neurology, University Hospital Muenster, Muenster, Germany

**Keywords:** dysphagia, myositis, antisynthetase syndrome, antisynthetase antibody, Jo-1, Jo-1 autoantibody, diagnostic algorithm, idiopathic inflamed myopathy

## Abstract

Dysphagia can be predominant or sole symptom of myositis. However, diagnostic evaluation is difficult in such cases. Here, we present evidence for dysphagia as sole manifestation of Jo-1 associated myositis. A 77-year-old patient suffering from isolated dysphagia was assessed by flexible endoscopic evaluation of swallowing, videofluoroscopy, high resolution esophageal manometry, whole body muscle MRI, electroneurographic and electromyographic examination, cerebrospinal fluid analysis, screening for autoantibodies, and body plethysmography. We detected isolated oropharyngeal dysphagia including a decreased pressure of the upper esophageal sphincter leading to cachexia in an anti-Jo-1 positive patient without any abnormalities in the other diagnostics. Immunosuppressive therapy with cortisone and azathioprine led to long-term improvement of dysphagia. This is the first report of isolated dysphagia as manifestation of Jo-1 associated myositis. Therefore, Jo-1 associated myositis should be considered as a possible differential diagnosis for isolated dysphagia. Typical signs for myositis in instrumental dysphagia assessment are presented.

## Introduction

Dysphagia is frequently reported in patients with myositis (1–6) with prevalence rates ranging from 30 to 72% (5, 6). Typical symptoms of myositis related dysphagia are coughing, choking, bolus-sticking in the pharynx and swallowing problems with dry and solid food consistencies (1, 5). Early diagnosis and specific therapeutic management of dysphagia is crucial as it can lead to aspiration pneumonia with respiratory failure, which is the leading cause of mortality in patients with myositis (1, 7). Dysphagia can occur as predominant or sole symptom of myositis: Oh et al. described dysphagia as the only manifestation of inclusion body myositis (8). Shapiro et al. reported 3 cases of isolated pharyngeal dysphagia with biopsies of the omohyoid and cricopharyngeus muscles showing inflammatory myopathy (9). In such cases, diagnostic evaluation is particularly difficult, as myositis-related dysphagia may also be present if laboratory or electrophysiological diagnostics remain unremarkable (10). The role of autoantibodies in the diagnosis of myositis is steadily increasing. Jo-1 antibodies are now part of the current EULAR diagnostic criteria alongside clinical criteria, CK blood level, and histological signs (11). Further, autoantibodies can be helpful in the classification of myositis (12). This biomarker driven development might also be beneficial in patients with unclear dysphagia. In this case-report we present evidence for dysphagia as isolated manifestation of Jo-1 associated myositis, which to the best of our knowledge has not been reported so far. In addition, we give an overview of the literature on the pathophysiology of dysphagia in myositis.

## Case Report

### Patient History

A 77-year-old patient was presented to our outpatient clinic due to progressive dysphagia. About 10 years ago, he noticed taste disturbances and discomfort in the mouth region, hindering his speech. During the last 3 years, he predominantly suffered from swallowing impairment. He reported of choking and hiccups in particular when eating solid food. In addition, he needed increasingly longer to finish a normal meal and had to extraordinarily concentrate on the eating process. As a consequence, he avoided eating in public. Recently, he was hospitalized due to an aspiration pneumonia. The patient did not take any medication. The detailed clinical examination revealed no fasciculations, muscle weakness, double vision, raynaud's phenomenon, mechanic's hands, skin rash or dyspnea, only a cachectic nutritional status due to dysphagia (size 1.8 m, weight 53 kg, BMI 16.4) was remarkable. There were no relevant pre-existing diseases. Cerebral MRI that had been performed before the presentation in our department showed no pathological findings.

### Diagnostic Procedures

**Figure 1:**
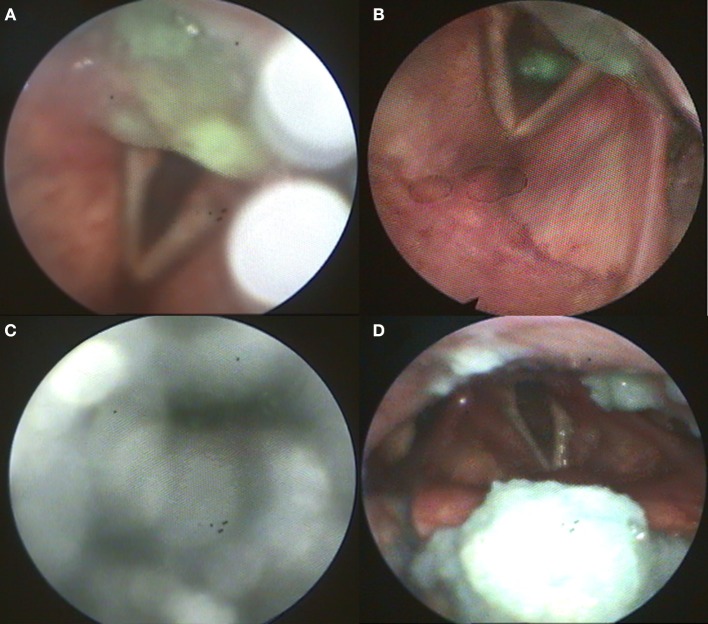
**(A)** Postdeglutitive residue of pudding; **(B)** Aspiration of pudding; **(C)** Weak white-out as sign for reduced pharyngeal contractility; **(D)** Postdeglutitive residue of bread.

Flexible endoscopic evaluation of swallowing (FEES) revealed severe oropharyngeal dysphagia with intradeglutitive aspiration and postdeglutitive residue in the vallecula und pyriform sinus (graded as severe for semisolid and solid consistencies according to the Yale Pharyngeal Residue Severity Scale (13) as a sign of weak pharyngeal constriction (Figure 1). The FEES tensilon test (14, 15) showed no improvement of dysphagia upon application of edrophonium. Videoflouroscopy of swallowing (VFSS) confirmed severe pharyngeal dysphagia with markedly restricted pharyngeal contraction and consecutive nasopharyngeal reflux as well as postdeglutitive aspiration due to considerable retention. Furthermore, there was a markedly delayed triggering of swallowing reflex with reduced hyolaryngeal elevation and predeglutitive aspiration, Rosenbek grade 7 (16, 17). High resolution esophageal manometry (HRM) showed a markedly reduced pressure of the upper esophageal sphincter with extended relaxation and recovery time and an amotile tubular esophagus. Functional dysphagia severity according to the FOIS-Score was 1 (18). Basic laboratory and CSF diagnostics were normal (no cytalbuminary dissociation, normal CK-blood level). CSF-screening for ganglioside antibodies was negative (Ganglioside Profile 2 IgG and IgM Euroline, Euroimmun; Lübeck, Germany). Whole body muscle MRI was unremarkable without edema or atrophy. The electroneurographic examination detected a length-dependent, axonal, sensitive polyneuropathy as incidental finding. Repetitive stimulation of the n. facialis on both sides with recording from the m. nasalis revealed no decrement. Repetitive stimulation of the n. ulnaris on the left side with recording from the m. abductor digiti minimi before and after 1-min maximum arbitrary innervation revealed no increment. In the electromyographic examination, isolated chronic neurological changes of the right hand muscles, but otherwise inconspicuous findings were observed (m. deltoideus on both sides: no pathological spontaneous activity, motor unit potential normally configurated, regular recruitment, dense interference; m. abductor pollicis brevis on both sides: no pathological spontaneous activity, motor unit potential on the right side partly with high amplitude, recruitment and interference on the right side slightly reduced; m. abductor digiti minimi on both sides: no pathological spontaneous activity, motor unit potential on the right side partly with high amplitude, recruitment and interference on the right side slightly reduced; m. vastus on both sides: no pathological spontaneous activity, motor unit potential normally configurated, regular recruitment, dense interference; m. tibialis anterior on both sides: no pathological spontaneous activity, motor unit potential normally configurated, regular recruitment, dense interference; paravertebral T8 on both sides: no pathological spontaneous activity; m. glossus on the left side: no pathological spontaneous activity). The antibody screening was positive for Jo-1 IgG (++) and negative for the other screened autoantibodies (ANA, Mi-2a, Mi-2b, TIF1g, MDA5, NXP2, SAE1, Ku, PM100, PM75, SRP, PL-7, PL-12, EJ, OJ, Ro52, Euroline, Euroimmun, Lübeck, Germany, and anti-HMG-CoA, MVZ Labor Volkmann, Karlsruhe, Germany). Also, no myasthenia antibodies could be detected (negative for AChR, titin, MuSK, and VGCC, Euroline, Euroimmun, Lübeck, Germany). Although the role of Jo-1-IgG in the pathophysiology of myositis is poorly understood, it is a highly reliable marker for idiopathic inflammatory myopathies (19). In the absence of signs for other diseases, this led to the diagnosis of Jo-1 associated myositis as underlying disease. Body plethysmography and x-ray-chest revealed no indication of pulmonary involvement.

### Therapy

A PEG tube was placed to ensure sufficient enteral nutrition. In addition, an intravenous cortisone therapy with 1 g methylprednisolone per day for 5 days followed by oral therapy with prednisolone and azathioprine was performed. After 4 months of therapy the swallowing function improved, and FEES showed a reduction in penetration and aspiration frequency and severity, lower levels of penetration and aspiration volume, and a more effective bolus clearance. The FOIS-scale had improved from 1 on admission to 3, so that a reinstitution of oral feeding parallel to the PEG nutrition was recommended (18). This improvement sustained in the long term (currently 12 months after beginning of therapy).

## Discussion

There are only few studies that investigated the pathophysiological characteristics of myositis related dysphagia. Ebert et al. claimed that myositis mainly affects the proximal esophageal skeletal muscle. They reported decreased upper sphincter pressure and absent pharyngeal contractions in manometry (3). Casal-Dominguez et al. also reported decreased upper esophageal sphincter pressure and failed waves in patients with polymyositis (20). Ertekin et al. showed that myositis mainly leads to pharyngeal dysphagia with prolonged phase of pharyngeal swallowing and weakness of the striated oropharyngeal muscles. Contrary to other manometry studies, they found that the cricopharyngeal sphincter was affected less frequently and showed both hyporeflexic and hyperreflexic states in myositis (2). In a study using VFSS, Langdon et al. reported delayed swallowing initiation, decreased hyolaryngeal excursion, pyriform residue and penetration (5). Consistent with these findings, Oh et al. also described impaired laryngeal elevation, pharyngeal pooling, disturbed tongue retraction and abnormal cricopharyngeal function (1).

Whether inflammatory myopathies constitute a homogeneous pathophysiological pathophysiological dysphagia entity or specific sub forms e.g., inclusion body myositis, dermatomyositis, and antisynthetase syndrome show their own specific pathophysiological characteristics largely remains an open question. Oh et al. did not find differences between inclusion body myositis, dermatomyositis, and polymyositis in VFSS findings (1). Casal-Dominguez et al. report that antisynthetase syndrome is associated with decreased and hypotonic lower esophageal sphincter pressure and conclude that the autoimmune reaction of this specific myositis entity may affect the smooth muscle of the esophageal body in particular and cause impairment in the lower esophageal sphincter (20). Inclusion body myositis seems to be rather associated with an increased pressure of the upper esophagus sphincter due to a relaxation deficit (21, 22) which manifests as pharyngeal muscle propulsion between C3 and C7 in VFSS (21). Typical findings of instrumental dysphagia assessment that may indicate myositis are shown in Table 1.

**Table 1:** Typical signs for myositis in instrumental dysphagia assessment.

**Flexible Endoscopic Evaluation of Swallowing:**
•*Reduced pharyngeal contractility (pharyngeal squeeze maneuver)*
•*Weak white-out*
•Pharyngeal residue
•Postdeglutitive penetration/aspiration
**Videofluoroscopy:**
•*Decreased hyolaryngeal elevation*
•*Pharyngeal muscle propulsions at C3-C7*
•Impairment of the upper or lower esophagus sphincter
•Pharyngeal residue
•Postdeglutitive penetration/aspiration
**High Resolution Manometry:**
•Impairment of the upper or lower esophagus sphincter

*Signs that are specific to one diagnostic procedure are printed in italics*.

Here, to our knowledge we present the first ever reported case of isolated dysphagia due to Jo-1 associated myositis: we found pharyngeal dysphagia with impairment of the upper esophagus similar to the impairment pattern commonly described in myositis. This case demonstrates that not only the smooth esophageal muscle (20) but also the striated muscle of the pharynx and upper esophagus can be affected by the Jo-1 associated autoimmune reaction. Based on our case report, we therefore recommend that myositis focused diagnostics including an autoantibody panel should be done in patients with unclear dysphagia that show typical sings for myositis in the instrumental dysphagia assessment.

## Limitations

There are only few studies that investigated the pathophysiological characteristics of myositis related dysphagia. The pathophysiological conclusions in this study must therefore be considered cautiously. According to the EULAR criteria, the probability of myositis in a patient with dysphagia and detection of Jo-1 antibodies is 80% (11). Myositis is therefore only the probable underlying disease. Since no muscle biopsy was performed, other diseases e.g., atypical IBM cannot be ruled out with certainty.

## Data Availability

The raw data supporting the conclusions of this manuscript will be made available by the authors, without undue reservation, to any qualified researcher.

## Ethics Statement

Written and informed consent for this case report and the related data and images was obtained by the patient prior to publication.

## Author Contributions

BL and TW were responsible for the preparation and conceptual design of the manuscript. TR was responsible for advising on antibody diagnostics. SA was responsible for the coordination and realization of the FEES examination. All other authors (PM, SS-K, and RD) were involved in the preparation and editing of the manuscript.

### Conflict of Interest Statement

The authors declare that the research was conducted in the absence of any commercial or financial relationships that could be construed as a potential conflict of interest.
